# Etoposide induced cytotoxicity mediated by ROS and ERK in human kidney proximal tubule cells

**DOI:** 10.1038/srep34064

**Published:** 2016-09-26

**Authors:** Hyeon-Jun Shin, Hyuk-Kwon Kwon, Jae-Hyeok Lee, Muhammad Ayaz Anwar, Sangdun Choi

**Affiliations:** 1 Department of Molecular Science and Technology, Ajou University, Suwon, 443-749, Korea; 2 Department of Materials Science and Engineering, Northwestern University, Evanston, Illinois 60208, USA

## Abstract

Etoposide (ETO) is a commonly used chemotherapeutic drug that inhibits topoisomerase II activity, thereby leading to genotoxicity and cytotoxicity. However, ETO has limited application due to its side effects on normal organs, especially the kidney. Here, we report the mechanism of ETO-induced cytotoxicity progression in human kidney proximal tubule (HK-2) cells. Our results show that ETO perpetuates DNA damage, activates mitogen-activated protein kinase (MAPK), and triggers morphological changes, such as cell and nuclear swelling. When NAC, a well-known reactive oxygen species (ROS) scavenger, is co-treated with ETO, it inhibits an ETO-induced increase in mitochondrial mass, mitochondrial DNA (*ND1* and *ND4*) copy number, intracellular ATP level, and mitochondrial biogenesis activators (*TFAM, PGC-1α* and *PGC-1β*). Moreover, co-treatment with ETO and NAC inhibits ETO-induced necrosis and cell swelling, but not apoptosis. Studies using MAPK inhibitors reveal that inhibition of extracellular signal regulated kinase (ERK) protects ETO-induced cytotoxicity by inhibiting DNA damage and caspase 3/7 activity. Eventually, ERK inhibitor treated cells are protected from ETO-induced nuclear envelope (NE) rupture and DNA leakage through inhibition of caspase activity. Taken together, these data suggest that ETO mediates cytotoxicity in HK-2 cells through ROS and ERK pathways, which highlight the preventive avenues in ETO-induced cytotoxicity in kidney.

ETO is one of the well-known chemotherapeutic drugs that inhibit topoisomerase II activity. It is commonly used alone or in combination with other anticancer agents to treat lung, ovarian, testicular, and various other cancers^1^. Nevertheless, the use of ETO has been limited due to its side effects on all of the normal tissues and organs, especially the kidney^2^,^3^,^4^. Recently, our studies examined ETO involvement in mediating DNA damage, cell cycle arrest, and ROS generation in HK-2 cells, which eventually leads to necrosis by a p53-mediated anti-apoptotic pathway. Inhibition of p53 significantly increased phosphorylation of ERK and accumulation of ROS in the mitochondria, and enhanced apoptosis and apoptosis-related morphological changes^5^. Moreover, cisplatin, a platinum-based chemotherapeutic drug, induced cytotoxicity in HK-2 cells by impairing glycolysis and tricarboxylic acid cycle, and generating ROS in the mitochondria. *N*-acetylcysteine (NAC), an ROS scavenger, inhibited cisplatin-induced cytotoxicity^6^.

Mitochondria are crucial for energy production, cellular metabolism, cell differentiation, and cell death^7^,^8^. They are also a main source of ROS, including superoxide (O_2_^−^) and hydroxyl and hydrogen peroxide (H_2_O_2_), under various stress responses^9^,^10^. Mitochondrial respiratory complexes I, II, and III are located in the inner mitochondrial membrane that releases O_2_^−^, which is used to produce H_2_O_2_ by superoxide dismutase 2 (SOD2) in the matrix. In contrast, mitochondrial respiratory complex III can be released in the intermembrane space and subsequently released into the cytoplasm by a voltage-dependent anion-selective channel (VDAC), where it mediates H_2_O_2_ production by superoxide dismutase 1 (SOD1)^11^. Generation of ROS causes nucleic acid and protein damage, and initiates an intracellular signal transduction pathway involved in development of cell death, cancer, and other diseases^12^,^13^,^14^. Furthermore, generation of ROS activates MAPK signaling pathways, which determine cell fate in different cell types^15^,^16^. In mammalian cells, the MAPKs primarily consist of ERK, c-Jun N-terminal kinase (JNK), and p38 MAPK. These kinases play a key role in controlling fundamental intracellular processes, such as cell proliferation, cell differentiation, survival, and cell death^17^,^18^,^19^. Previous studies showed that cisplatin induces apoptotic cell death in ovarian cancer cells through activation of JNK and p38^20^, and activation of ERK in cervical cancer cells^21^. Generation of ROS and activation of MAPKs occurs in response to cellular stress induced by chemotherapy drugs, systemic diseases and environmental toxins, which are closely associated with nephrotoxicity^22^,^23^,^24^,^25^. However, ETO induction of ROS and MAPK pathways in nephrotoxicity is not fully understood.

Cell death is generally classified as necrosis and apoptosis based on their morphological characteristics; cell swelling and plasma membrane ruptures are related to necrosis, whereas cell shrinkage and formation of apoptotic bodies are characteristics of apoptosis^26^. Specially, apoptotic cell death is characterized by destruction of the nuclear envelope (NE) and fragmentation of DNA and protein^27^. The NE protects genetic information by separating the nucleus from the cytoplasm and controls nucleocytoplasmic transport of molecules such as protein and RNA^28^,^29^. The NE consists of an inner nuclear membrane (INM) and outer nuclear membrane (ONM), whereas the nuclear pore complexes (NPCs) are embedded in the double-membranes^30^,^31^. In nucleus, intermediate filament proteins A- and B-type lamins connect the INM with DNA, provide NE structural integrity, and DNA stabilization^32^,^33^. However, when DNA damaging chemicals induce apoptosis, NPC and INM proteins are cleaved by activated caspases. In previous studies, NE disruption was indirectly confirmed by fluorescence intensity measurement using a nuclear targeting dye, and NE protein degradation was demonstrated by western blots^34^,^35^,^36^.

Here, we demonstrate that ETO-induced cytotoxicity in HK-2 cells is executed through ROS generation and ERK activation. Moreover, ETO causes NE rupture and DNA leakage mediated by ERK activation of caspase. The resulting topographical changes were directly measured using atomic force microscopy (AFM).

## Results

### ETO induced DNA damage, MAPKs activation, and nuclear swelling

**Figure 1:**
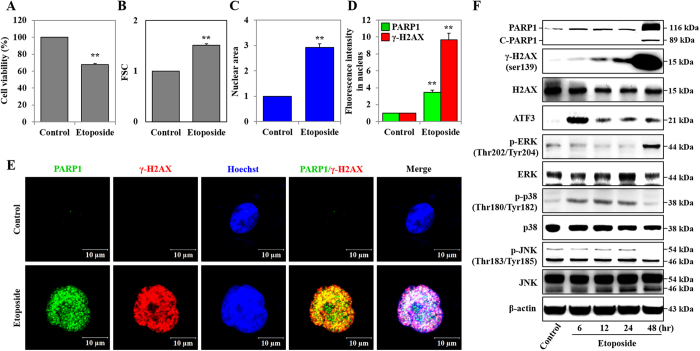
Etoposide induced DNA damage, MAPKs activation, and nuclei swelling. HK-2 cells were treated with Etoposide (50 μM) for 48 hours. (**A**) Cell viability was measured using an MTT assay. (**B**) Cell size was measured by forward scattered light (FSC) unit and detected using a FACS system. (**C**) The nucleus area was measured by Hochest33258 staining, and at least 3,000 cells were detected using Cellomics ArrayScan VTI HCS Reader. (**D**) The expression of γ-H2AX^ser139^ and PARP1 in the nuclei from at least 3,000 cells was measured by immunofluorescence staining, and detected using Cellomics ArrayScan VTI HCS Reader system. (**E**) The nuclear swelling was observed by Hoechst 33258 staining (blue color) and expression of γ-H2AX^ser139^ (red) and PARP1 (green) in the nuclei was determined by immunofluorescence staining and detected using confocal microscopy (scale bar represents 10 μm). (**F**) The protein expression levels of PARP1, C-PARP1, ATF3, p-ERK^Thr202/Tyr204^, ERK, p-p38^Thr180/Tyr182^, p38, p-JNK^Thr183/Tyr185^ and JNK in etoposide (50 μM) treated cells over time were analyzed by western blot. β-actin was used as a loading control. All data shown represent the mean ± SEM of three independent experiments (*P < 0.05, **P < 0.01).

HK-2 cells were exposed to 50 μM of ETO for 48 hours, and cell viability was assessed with the 3-[4,5-dimethylthiazol-2-yl]-2,5-diphenyltetrazolium bromide (MTT) assay. There was a relevant reduction in viability of ETO-treated cells, which decreased by 32.4% when compared to the control group (Fig. 1A). Observed morphological changes in cells undergoing necrotic death are cellular and nuclear swelling, while cell shrinkage and nuclear fragmentation are exhibited in apoptotic cells^26^,^37^. We used forward scatter units (FSC; cell size) and Cellomics ArrayScan VTI HCS Reader to show that ETO induces cell and nuclear swelling compared to untreated HK-2 cells (Fig. 1B,C). DNA damage induced by chemotherapeutic drugs is reported to increase phosphorylation of H2A histone family member X (γ-H2AX), and cleaved-poly (ADP-ribose) polymerase 1 (C-PARP1)^38^,^39^. Moreover, the exposure of cells to various types of stress, such as oxidative, ER, and genotoxicity stresses, increases the expression of activating transcription factor 3 (ATF3)^40^. Our previous study reported that etoposide-induced DNA damage perpetuates through DNA damage response (DDR) proteins such as PARP1, γ-H2AX, BRCA1, 53BP1 and ATM in HK-2 cells^5^. As shown in Fig. 1D,F, ETO time-dependently induced expression of γ-H2AX, PARP1 (116kDa), and C-PARP1 (89kDa). Additionally, expression of γ-H2AX and PARP1 was significantly increased in the nuclei of at least 3,000 cells (Fig. 1D,F). Similarly, confocal microscopy images show that ETO treatment induces nuclear swelling and nuclear expression of γ-H2AX and PARP1 in HK-2 cells compared to untreated samples (Fig. 1E). ETO induces phosphorylation of ERK (p-ERK) at 48 hours and phosphorylation of p38 (p-p38) at 6–24 hours, but the level of phosphorylated p38 (p-p38) is reduced at 48 hours. The phosphorylated JNK (p-JNK) level is not significantly altered at 6–24 hours but it has been reduced significantly at 48 hours (Fig. 1F).

### ETO induced DNA damage, mitochondrial biogenesis, and cytotoxicity through ROS generation

**Figure 2:**
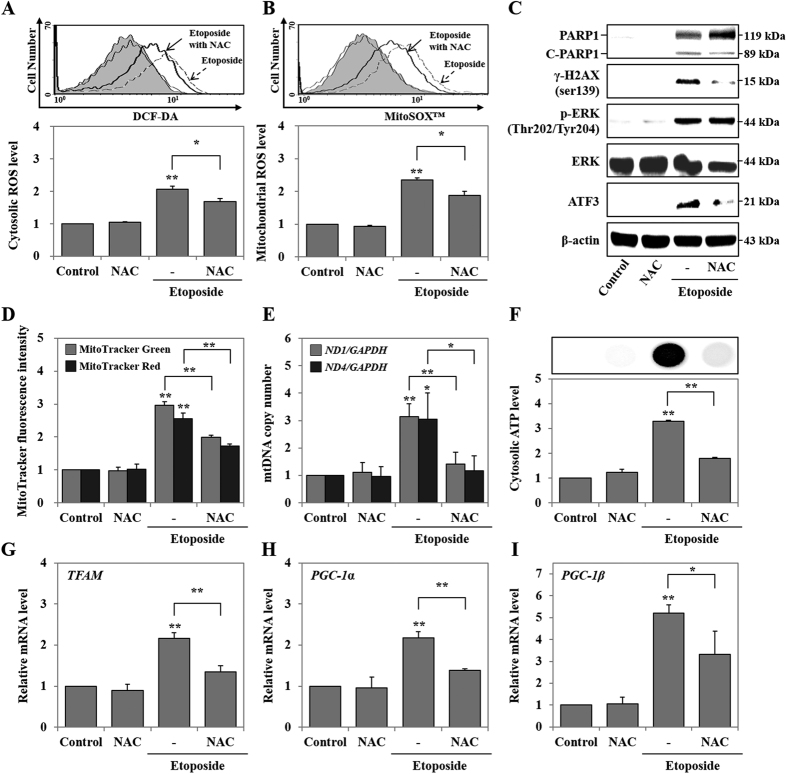
Etoposide induced DNA damage and mitochondrial biogenesis through ROS generation. HK-2 cells were pre-treated with NAC (5 mM) for 1 hour and then treated with etoposide (50 μM) for 48 hours. (**A**,**B**) Generation of ROS in cytosol (**A**) and mitochondria (**B**) were measured by DCF-DA and MitoSOX™ fluorescence intensity of cells using a FACS system, respectively. (**C**) The protein expression levels of PARP1, C-PARP1, p-ERK^Thr202/Tyr204^, ERK, and ATF3 were measured using western blot. β-actin was used as a loading control. (**D**) Mitochondrial mass and respiration were determined by MitoTracker Green FM and MitoTracker Red CMXRos, respectively, using the FACS system. (**E**) Mitochondrial DNA copy number was measured in mitochondrial-encoded genes, such as *ND1* and *ND4,* using RT-PCR and normalized to *GAPDH* copy number. (**F**) The cytosolic ATP level was measured by CellTiter-Glo^®^ Luminescent Cell Viability Assay using a luminescence microplate reader; luminescence intensity is presented in the image. (**G–I**) The expression levels of mitochondrial biogenesis-related genes, such as *TFAM, PGC-1α*, and *PGC-1β*, were measured using RT-PCR and normalized to *GAPDH*. All data shown represent the mean ± SEM of three independent experiments (*P < 0.05, **P < 0.01).

**Figure 3:**
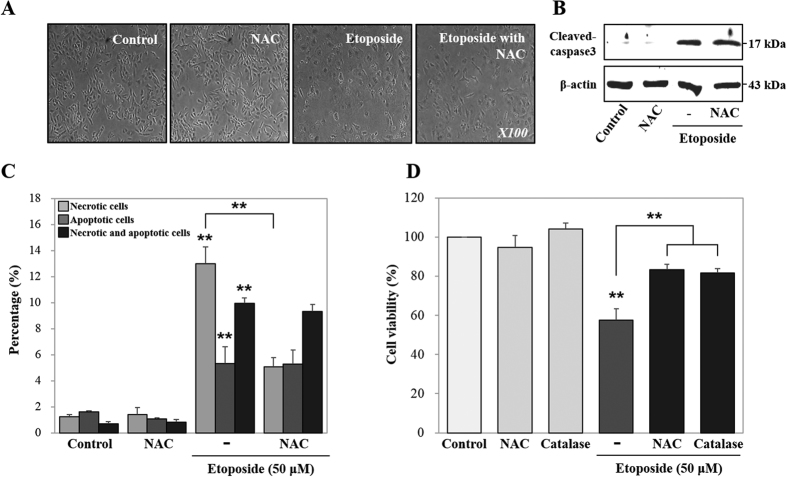
Etoposide induced cell swelling and cytotoxicity through ROS generation. HK-2 cells were pre-treated with NAC (5 mM) for 1 hour and then treated with etoposide (50 μM) for 48 hours. (**A**) Cell morphology was observed using phase contrast microscopy (original magnification *X100*). (**B**) The protein expression levels of cleaved-caspase3 detected by western blot were quantified using β-actin as a loading control. (**C**) Analysis of cell death including necrosis, apoptosis and necrosis with apoptosis, as measured using Annexin V and propidium iodide (PI) double staining. Necrotic and/or apoptotic cells were detected by FACS analysis. Histogram indicates the percentage of fluorescence positive cells. (**D**) HK-2 cells were pre-treated with NAC (5 mM) and catalase (100 U/ml) for 1 hour and then treated with etoposide (50 μM) for 48 hours. Cell viability was measured using MTT assay. Data shown represent the mean ± SEM of three independent experiments (*P < 0.05, **P < 0.01).

Chemotherapeutic drug induction of oxidative stress, such as ROS generation, has been reported to inflict cytotoxicity on diverse cellular molecules, such as DNA, lipid, and protein^24^. DNA-damaging agents, such as cisplatin, etoposide or doxorubicin, catalyze the formation of ROS^41^. Our previous study reported that etoposide induced cytosolic NO, NO secretion, cytosolic ROS and mitochondrial ROS, and the expression levels of cyclooxygenase 2 and iNOS after 48 hours of treatment in HK-2 cells^5^. Therefore, we examined the effect of oxidative stress in ETO treated HK-2 cells by co-treating with NAC, a known ROS scavenger, for 48 hours. NAC reduces ETO-induced ROS generation in the cytosol and mitochondria when compared with cells treated with ETO alone (Fig. 2A,B). Moreover, NAC inhibits ETO-induced expression of γ-H2AX and ATF3, but does not significantly affect PARP1 (119 kDa), C-PARP1 (89 kDa), and p-ERK expression when compared with ETO treatment alone (Fig. 2C). Further, to examine the effect of ETO and/or NAC on mitochondrial dynamics at 48 hours, we used MitoTracker Red and MitoTracker Green double staining to measure modulation of mitochondrial mass and respiration in HK-2 cells treated with either ETO or ETO in combination with NAC. MitoTracker Red detects mitochondrial respiration through mitochondrial membrane potential, but MitoTracker Green can detect mitochondrial mass independent of the mitochondrial membrane potential. Figure 2D shows that fluorescence intensities of MitoTracker Red and Green are simultaneously increased in ETO treated cells, while decreased in NAC-treated cells. Subsequently, we investigated mRNA expression levels of mitochondrial DNA (mtDNA) [NADH dehydrogenase subunit 1 and 4 (*ND1* and *ND4*)] as well as mitochondrial biogenesis inducing factors including transcription factor [nuclear-encoded transcription factors (*TFAM*)] and coactivators [peroxisome proliferator-activated receptor-gamma coactivator-1 α and β (*PGC-1α* and *PGC-1β*)]^42^. The data show that NAC inhibits ETO-induced expression of mtDNA (*ND1* and *ND4*), transcription factor (*TFAM*), and coactivators (*PGC-1α* and *PGC-1β*) (Fig. 2E,G–I). The production of cytosolic ATP was induced by ETO; however, diminished ATP level was observed when cells were co-treated with ETO and NAC (Fig. 2F). Furthermore, ETO and NAC co-treated cells show mediocre cell swelling, but the expression level of cleaved caspase3 was not significantly altered (Fig. 3A,B). ETO treated cells displayed increased necrotic cells (from 1.2% ± 0.2% to 13.0% ± 1.3%) and apoptotic cells (from 1.6% ± 0.2% to 5.3% ± 0.4%); however, when co-treated with NAC, the increase of necrotic cells (from 1.2% ± 0.2% to 5.1% ± 0.7%) and apoptotic cells (from 1.6% ± 0.2% to 5.3% ± 0.6%) was lower as compared to control. Eventually ROS inhibition significantly decreased the percentage of necrotic cells (67.4%), but did not alter the percentage of apoptotic cells compared to ETO alone (Fig. 3C). Besides, we confirmed the increased cell viability in NAC or catalase co-treated cells compared to ETO treated cells (Fig. 3D). Consequently, ROS-induced DNA damage, mitochondrial biogenesis, and cytotoxicity are reversed when NAC is part of the treatment regimen; however, it does not affect ERK activation and apoptotic cell death.

### ETO induced DNA damage and cytotoxicity through ERK activation

**Figure 4:**
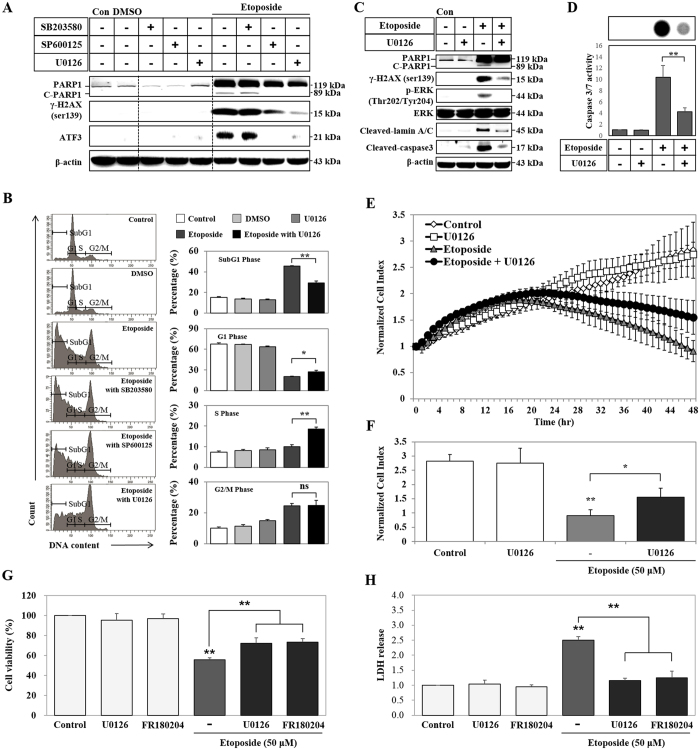
Etoposide induced DNA damage and cytotoxicity through ERK activation. (**A**) HK-2 cells were pre-treated for 1 hour with different MAPK inhibitors (10 μM of SB203580, 20 μM of SP600125 and 20 μM of U0126), and then treated with DMSO (vehicle control) or 50 μM of etoposide. The protein expression levels of γ-H2AX^ser139^, PARP1, C-PARP1 and ATF3 detected by western blots. β-actin was used as a loading control. HK-2 cells were pre-treated with U0126 (20 μM) for 1 hour and then treated with etoposide (50 μM) for 48 hours. (**B**) DNA content in different cell cycle stages was determined by propidium iodide (PI) staining using FACS analysis. This method gives the percentage of cells in each phase as shown in the histogram. (**C**) The protein expression levels of γ-H2AX^ser139^, PARP1, C-PARP1, p-ERK^Thr202/Tyr204^, ERK, cleaved-lamin A/C, and cleaved-caspase3 detected by western blot. β-actin was used as a loading control. (**D**) Caspase 3/7 activity was measured in a Caspase-Glo 3/7 assay using a luminescence microplate reader; luminescence intensity is shown in the image. (**E**) Proliferation of HK-2 cells treated with either U0126, or etoposide or etoposide in combination with U0126 was measured using the xCELLigence system in real-time at 1 hour intervals for 48 hours. HK-2 cells were seeded on E-Plate 16 devices and grown overnight to form monolayers. The next day, cells were pre-treated with U0126 (20 μM) for 1 hour followed by treatment with etoposide (50 μM). Cell proliferation was measured by changes in electrical impedance (cell index) on the surface. (**F**) Histogram of cell proliferation measured by cell index at 48 hours. (**G**) HK-2 cells were pre-treated for 1 hour with U0126 (20 μM) and FR180204 (10 μM), and then treated with 50 μM of etoposide. Cell viability was measured using MTT assay. (**H**) Cytotoxicity was analyzed using LDH assay. Data shown represent the mean ± SEM of three independent experiments (*P < 0.05, **P < 0.01).

MAPK pathways have been studied in relation to cell proliferation, survival, and apoptosis in response to treatment with chemotherapeutic drugs^43^. Accordingly, we examined the effect of MAPKs on ETO-induced cytotoxicity using pharmacological MAPKs inhibitors SB203580 (p38 inhibitor), SP600125 (JNK inhibitor), U0126 (inhibits MEK1/2; ERK inhibitor) at 48 hours of treatment. Expression levels of γ-H2AX, PARP1 (119 kDa), C-PARP1 (89 kDa), and ATF3 are decreased by JNK and ERK inhibitors, while their expression levels remain unaffected in SB203580 treated cells (Fig. 4A). Similarly, the ERK inhibitor has a profound effect on the expression of γ-H2AX and C-PARP1 (89 kDa) stimulated by ETO (Fig. 4A,C). Lamins are components of the nuclear lamina, a fibrous layer on the nucleoplasmic side of the inner nuclear membrane, which is thought to provide a framework for the NE and may also interact with chromatin. Lamin A and C are present in equal amounts in the lamina of mammals. They play an important role in chromatin organization, nuclear assembly, and NE dynamics^44^. The NE proteins, lamin A/C, are cleaved by ETO treatment, but ERK inhibition blocks this cleavage (Fig. 4C). Moreover, ETO-induced activation of caspase3 and caspase 3/7 is significantly decreased by inhibiting ERK (Fig. 4C,D). Propidium iodide (PI) staining indicates that cells treated with ETO have a higher percentage of DNA in SubG1 (indicates apoptosis) and G2/M phases, but a lower percentage in G1 phase. On the contrary, ERK inhibition decreases the percentage of DNA in SubG1 phase and increases the percentage of DNA in G1 and S phase; it does not have any effect on DNA content in G2/M phase when compared with ETO treatment in the absence of the inhibitor (Fig. 4B). To identify the role of ERK in ETO-induced DNA damage, we next examined cell viability in a real-time manner using the xCELLigence RTCA. The xCELLigence RTCA system measures cell viability, proliferation, invasion, and migration by electrical impedance (cell index represented by a value) using a gold microelectrode pattern at real-time^45^,^46^. The results show that inhibition of ERK significantly protects cells, whereas ETO-treated cells lose their proliferation at 48 hours (Fig. 4E,F). FR180204 is a novel ERK-selective inhibitor that can permeate the cell, and has been reported to inhibit ERK1 and ERK2^47^. U0126 and FR180204 co-treated with ETO increased cell viability and decreased cytotoxicity compared with ETO treated cells (Fig. 4G,H). Thus, ETO induced DNA damage and cytotoxicity is perpetuated by ERK activation.

### ETO induced NE ruptures through ERK-mediated caspases activation

**Figure 5:**
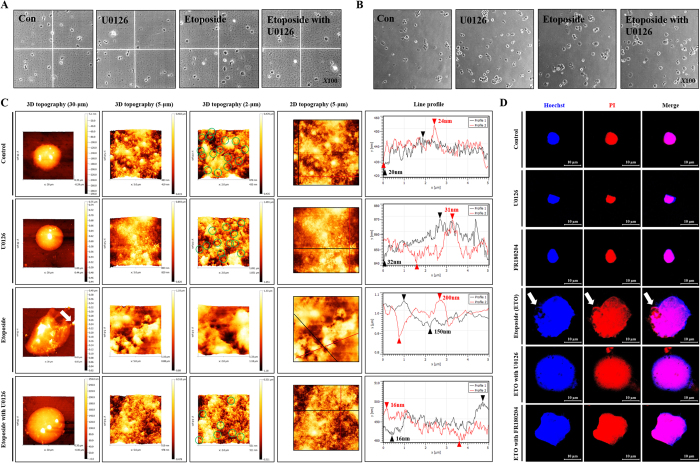
Etoposide induced nuclear envelope ruptures through ERK activation. HK-2 cells were pre-treated with U0126 (20 μM) or FR180204 (10 μM) for 1 hour followed by treatment with etoposide (50 μM) for 48 hours. Nuclei were extracted from the cells using NE-PER^®^nuclear and cytoplasmic extraction reagents. (**A**) Nuclear morphological changes were measured using a hematology analyzer and detected by microscopy (original magnification *X100*). (**B**) Nuclei were extracted, seeded and attached on culture dish for 15 minutes, where morphological changes such as nucleus swelling were measured using microscopy (original magnification *X100*). (**C**) Topography of NE was measured using an AFM probe system. Images shown are representative of 3-D topography at 30, 10, and 2 μm scales. (**D**) Nuclear extracts were seeded on coverslips for 15 min and stained with nucleus targeting dyes such as Hoechst 33258 (blue color) and propidium iodide (red color) that were detected using confocal microscopy. Arrows indicate the DNA leakage.

Apoptotic cell death presents unique characteristics, such as NE and DNA fragmentation, by activation of caspases in response to pro-apoptotic stimulators^27^. In this study, we identified that ETO treatment triggers caspase 3/7 activity that can be halted by the ERK inhibitor. Previously, we confirmed that the AFM probe by the Langmuir-Blodgett technique supported high-resolution imaging to accurately identify the morphology of nanostructures (nano-porous alumina membrane) and biological materials (plasmid DNA)^48^. Recently, we used a CNT/AFM technique to document that chemotherapy drugs, such as ETO, rupture the plasma membrane in HK-2 cells^5^. Moreover, we used the CNT/AFM technique to show for the first time that ETO induces NE ruptures through activation of caspases in HK-2 cells^49^. Based on these results, we hypothesized that the disruption of the NE by ETO stimulates activation of caspases, while inhibition of ERK suppresses disruption of the NE. We used NE-PER Nuclear and Cytoplasmic Extraction Kit for accurate and secure nuclear extraction. The extraction kit contains Cytoplasmic Extraction Reagent I and II (CER I and II), and Nuclear Extraction Reagent (NER). The addition of the first two reagents to cell pellet causes cell membrane disruption and the release of cytoplasmic contents. After recovering the intact nuclei from the cytoplasmic extract by centrifugation, the nuclear proteins are extracted using the third reagent. For isolation of nuclei, we only used CER I and II. CER I causes cell membrane disruption, but not disruption of the NE, while CER II inhibits the activity of CER I. This extraction system has already been published^49^. Accordingly, we extracted nuclei from HK-2 cells treated with U0126, ETO, or ETO in combination with U0126 or FR180204, and then evaluated nuclear integrity through hemocytometer analysis. The extracted nuclei from ETO-treated and ETO/U0126-treated cells definitely demonstrate swelling when compared with untreated and U0126-treated cells; the visual observations are consistent with confocal microscopy images shown in Fig. 1E (Fig. 5A). The extracted nuclei were seeded in tissue culture plates for 15 minutes to enable their attachment. Similar to Fig. 5A, nuclei extracted from ETO-treated and ETO/U0126-treated cells display nuclear swelling (Fig. 5B). Moreover, supernatants extracted from the nuclei of ETO-treated cells are turbid owing to nuclear disintegration, whereas relatively low turbidity is observed in the ETO/U0126-treated cells. Since phase contrast microscopy has limited observation for nuclear envelope, we directly measured NE topography from the extracted nuclei using AFM analysis. The results demonstrate that the nuclei from HK-2 cells are round shaped with a large number of intact NPC (green circles at 2-μm scales). However, nuclei from ETO-treated cells have nuclear swelling with an irregular nuclear shape, released-DNA (DNA leakage), and a number of NE ruptures with disappearance of NPC when compared with untreated and U0126 treated HK-2 cells. Furthermore, using line profile analysis, untreated (control) and U0126 treated HK-2 cells demonstrate a lower average of roughness (20–30 nm), but ETO treated cells demonstrate a significantly higher average of roughness (100–200 nm). On the contrary, nuclei from ERK-inhibited HK-2 cells suppress ETO-induced DNA leakage and NE ruptures, but nuclear swelling remains unaffected (Fig. 5C). Additionally, we confirmed nuclear swelling, NE rupture and DNA leakage through confocal micoroscopy using Hoechst 33258 and PI staining. Consequently, ETO-treated cells showed nuclear swelling with an irregular nuclear shape and DNA leakage (white arrow). ERK-inhibited cells (by U0126 and FR180204) protected nuclear shape and DNA leakage, but did not affect nulear swelling. (Figure 5D). Therefore, ETO-induced nuclear and NE damages are mediated by activation of caspase through ERK.

## Discussion

It is well established that cisplatin mediates key factors in cell physiology, including ROS generation and MAPK activation, to result in nephrotoxicity, which is defined by reduction of blood flow and glomerular filtration rate of the kidneys^50^. Recently, our studies demonstrated that cisplatin induces cytotoxicity mediated by mitochondrial ROS generation in HK-2 cells^6^. Similarly, ETO also induces ROS generation and ERK activation during necrosis. Inhibition of p53 leads to enhanced ERK activation and ROS accumulation in the mitochondria during ETO-induced apoptosis in HK-2 cells^5^. Doxorubicin-induced cytotoxicity is protected by inhibition of ERK in HK-2 cells^51^. However, the function of ERK activation and ROS generation in the ETO-mediated nephrotoxicity is not fully understood. In this study, we demonstrated the mechanism by which ETO-induced cytotoxicity mediates ROS generation and ERK activation.

Our results show that ETO induces DNA damage and ROS generation that triggers necrotic morphology, such as nucleus and cell swelling. An ROS scavenger was used to investigate the function of ROS generation in the ETO-induced cytotoxicity. We show that an ROS scavenger inhibits ETO-induced mitochondrial biogenesis through reduction of mitochondrial mass with respiration, cytosolic ATP, mitochondrial DNA copy number and mitochondrial biogenesis inducing factors, but does not affect the activation of ERK (p-ERK). Finally, the ROS scavenger protects the cells from ETO-induced necrosis and necrotic-like morphological changes. Mitochondrial biogenesis is regulated by nuclear-encoded transcription factors (TFAM) and coactivator proteins (PGC-1α and PGC-1β) that are activated by chemicals that stimulate DNA damage; they contribute to antioxidant defense processes^42^,^52^,^53^. For example, H_2_O_2_-induced mitochondrial biogenesis and respiration via upregulation of *PGC-1α* and *PGC-1β* expression, eventually prevents oxidative damage and H_2_O_2_-mediated cell death in neural cells^54^. In vascular endothelial cells, PGC-1α reduces accumulation of ROS and induced mitochondrial membrane potential that suppresses apoptosis caused by oxidative stress^55^. Mitochondrial biogenesis, stimulated by NO/cGMP in the brain and kidney, is associated with increased mitochondrial respiration, which results in enhanced ATP production^56^. Additionally, our recent study showed that increased cytosolic ATP, produced through mitochondrial hyper-activation, can contribute to necrosis^57^. Based on these results, we examined the mechanisms by which ETO-induced ROS generation enhances biogenesis of mitochondria to prevent oxidative stress, but does not affect ERK activation. Moreover, ROS enhanced necrosis and increased levels of cytosolic ATP mediated by mitochondrial biogenesis can contribute to necrosis.

ERKs are widely expressed protein kinases that regulate various functions, including cell differentiation, meiosis, and mitosis. ERK1 and ERK2 pathways can be activated by numerous stimuli, such as ligands for heterotrimeric G protein-coupled receptors, growth factors, cytokines, viral infection, and transforming agents^58^. Previously, our studies reported that HK-2 cells co-treated with ETO and p53 inhibitor have enhanced ERK activation and caspase activity as compared to cells treated with ETO alone; this leads to apoptosis^5^. Moreover, the pharmacological pan-caspase inhibitor, z-VAD, almost completely inhibits ETO-induced NE rupture and DNA leakage in HK-2 cells^49^. Our results show that ETO associated with ERK activation increases the number of PI and Annexin V positive cells. Additionally, the ERK inhibitor reduces DNA damage, caspase activity, C-PARP1, cleaved-lamin A/C, NE rupture, and DNA leakage, which altogether undermine the ETO cytotoxicity. Furthermore, 3 dimensional (3-D) nanoscale topography established that direct morphological changes, such as nuclear swelling, DNA leakage, NPC, and NE rupture including depth, width and volume, have been ameliorated. Measurement of morphology is crucial to confirm the observation of cytomorphological changes of cells so that a better understanding of the cell death processes, such as necrosis and apoptosis, can be obtained. Generally, necrotic cell death demonstrates cell swelling and plasma membrane ruptures, whereas apoptotic cell death is characterized by cell shrinkage and apoptotic body formation. These morphological characteristics can usually be measured by scanning electron microscope^26^. When apoptosis occurs as a result of chemical induced DNA damage, nuclear shape and NE disruption are generally detected by the fluorescence intensity of nuclear targeting dye and/or expression of NE proteins^34^,^35^,^36^. However, these techniques have a limitation due to indirect capturing of the morphological effects. Additionally, these techniques are not ideal for capturing NE topographical changes. Recently, we used AFM to document the morphological changes, including necrosis and apoptosis, stimulated by DNA damaging agents such as ETO and doxorubicin^5^,^57^. Moreover, based on nuclear and NE topography dynamics, the process is classified as necrosis or apoptosis, which can be measured directly by AFM after nuclear extraction. AFM analysis shows that necrosis is perpetuated through nuclear swelling, but NE topography is not affected. Contrary to this, apoptosis imparts NE rupture and DNA leakage by caspase activation^49^. Based on these results, we believe that ETO-induced ERK activation leads to caspase activation independent of ROS generation. Later, ERK-induced caspase activation, which promotes NE rupture and DNA leakage through cleavage of NE proteins, eventually leads to apoptosis.

**Figure 6:**
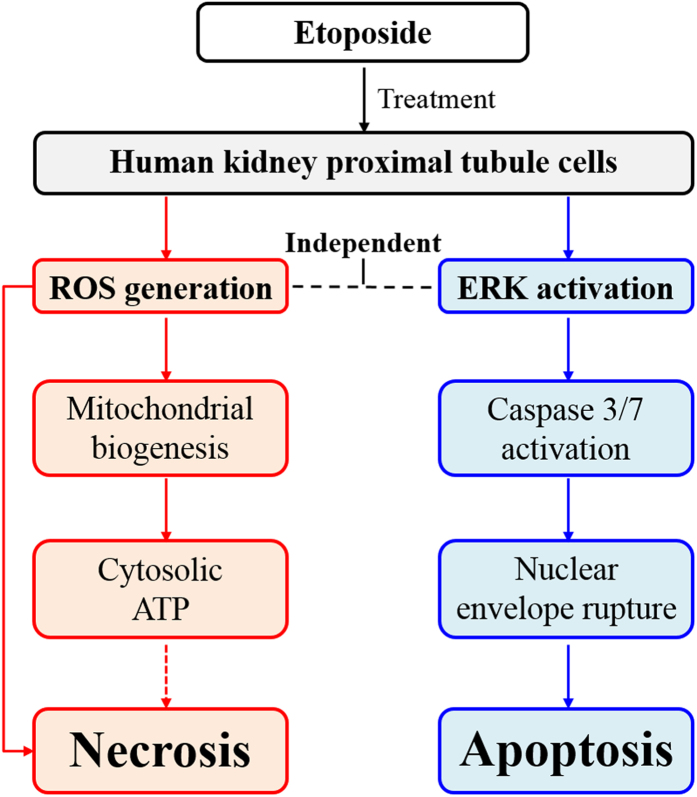
Overview of the mechanism underlying ROS- and ERK-mediated cytotoxicity. Etoposide treatment triggers ROS generation and ERK activation in HK-2 cells. ROS promotes mitochondrial biogenesis and cytosolic ATP induction, which eventually enhance necrosis, but not apoptosis. Whereas, ERK activation causes caspase 3/7 activation that in turn ruptures the nuclear envelope, which eventually induces apoptosis. Furthermore, ERK activation is independent of ROS generation.

Taken together, ETO stimulates ROS generation that leads to necrosis, whereas, ROS independent ERK activation is a crucial factor for induction of apoptosis through caspase activation in HK-2 cells (Fig. 6); these data provide a better understanding of the nephrotoxicity mechanism. Moreover, we demonstrate that a simple method using AFM analysis can recognize the topographical changes of the NE associated with necrosis and apoptosis. This technique is expected to be broadly applicable in various cancer and morphology-related studies.

## Methods

### Cell culture and treatment

Human kidney proximal tubule cell line (HK-2) was purchased from American Type Culture Collection (ATCC, Manassas, VA, USA) and was maintained in RPMI1640 medium supplemented with 1% penicillin/streptomycin and 10% fetal bovine serum (Thermo Fisher Scientific Inc., Waltham, MA, USA). Cells were incubated at 37 °C in an atmosphere of 5% CO_2_ (Thermo Fisher Scientific Inc.). Cells (4 × 10^5^) were seeded into a 6-cm dish (SPL Life Sciences., Pochun, South Korea) and incubated overnight at 37 °C in an atmosphere of 5% CO_2_. The medium was changed and etoposide (Sigma-Aldrich Co. LLC, St. Louis, MO, USA) was added as indicated. For MAPK inhibition experiments, cells were pre-treated for 1 hour with inhibitors of various signaling pathways: 10 μM SB203580 (p38MAPK inhibitor), 20 μM SP600125 (JNK inhibitor), 20 μM U0126 (inhibitors of MEK1 and MEK2, thus ERK) (Sigma-Aldrich Co. LLC), or 10 μM FR180204 (ERK 1/2 inhibitor) (Santa Cruz). For ROS inhibition experiments, cells were pre-treated with NAC or catalase (Sigma-Aldrich Co. LLC).

### Analysis of cell viability

The MTT assay [(1-(4,5-Dimethylthiazol-2-yl)-3,5-diphenylformazan, Sigma-Aldrich Co. LLC (St. Louis, MO, USA)] was used to determine cell viability. HK-2 cells (5 × 10^3^/well) were seeded into 96-well plates (BD Biosciences., San Diego, CA, USA), grown overnight, and treated with etoposide for 48 hours. Next, treated and untreated HK-2 cells were incubated with an MTT solution (100 μl/well) for 3 hours in a humidified atmosphere containing 5% CO_2_ at 37 °C. After incubation, the MTT solution was removed and DMSO solution (100 μl/well) was added for 30 minutes. Cell viability was measured using a microplate spectrophotometer system (Molecular Devices Inc., Sunnyvale, CA, USA) at 540 nm.

### Analysis of cell size

HK-2 cells (4 × 10^5^ cells/6cm culture dish) were grown overnight and treated with etoposide for 48 hours. Treated and untreated HK-2 cells were collected and washed with 3 ml of phosphate buffered saline (PBS) and centrifuged at 200× *g* for 5 min at 4 °C. Cell size was measured for at least 1 × 10^5^ cells by forward scattered light (FSC) unit analysis using FACS Aria™ III with Diva™ software (BD Biosciences).

### Analysis of Cellomics ArrayScan HCS Reader

HK-2 cells (1 × 10^4^/well) were grown overnight in a clear black 96-well plate (Greiner Bio-One., Frickenhausen, Germany) and treated with etoposide for 48 hours. Untreated and treated cells were fixed with 3.7% formaldehyde (15 minutes), permeabilized with 0.2% Triton X-100 (15 minutes) and blocked with 5% FBS (1 hour). Primary antibodies (1:1000) specific for γ-H2AX and PARP1 (Santa Cruz Biotechnology, Inc., Dallas, TX, USA) were added to the cells and incubated for 1 hour at room temperature. Following washes to remove the primary antibodies, mouse or rabbit secondary antibodies, conjugated to Alexa Fluor 488 or 546 (Invitrogen, Carlsbad, CA, USA) (1:1000), were added to each well and incubated for 1 hour at room temperature. Nuclei were stained with Hoechst 33258 dye (5 μM, 15 minutes) (Sigma-Aldrich Co. LLC). Fluorescence intensity and nuclear area for 3000 cells were analyzed by Cellomics ArrayScan HCS Reader (Thermo Fisher Scientific Inc.) and ArrayScan^®^ VTI (600 series) Version 6.6.1.3 software (Thermo Fisher Scientific Inc.).

### Analysis of confocal microscopy

HK-2 cells (4 × 10^5^ cells/6 cm culture dish) were grown overnight and treated with etoposide for 48 hours. Untreated and treated HK-2 cells were fixed with 3.7% formaldehyde (15 minutes), permeabilized with 0.2% Triton X-100 (15 minutes) and blocked with 5% FBS (1 hour). Primary antibodies (1:1000, 1 hour) to γ-H2AX and PARP1 (Santa Cruz Biotechnology, Inc.) and mouse or rabbit secondary antibodies conjugated with Alexa fluor^®^ 488 or 546 (Invitrogen) (1:1000) were added to the culture dish and incubated for 1 hour at room temperature. Nuclei were stained with Hoechst 33258 dye (5 μM, 15 minutes, Sigma-Aldrich Co. LLC). The fluorescence intensity of Alexa Fluor 488 and 546 were measured by confocal microscopy (LSM-700, Carl Zeiss MicroImaging, Germany), and analyzed by Zen 2009 software (Carl Zeiss MicroImaging).

### Western blot

HK-2 cells (4 × 10^5^ cells/6 cm culture dish) were grown overnight and treated with ETO at the indicated concentrations. Proteins were extracted using M-PER Mammalian Protein Extraction Reagent supplemented with protease and phosphatase inhibitor cocktails (Thermo Fisher Scientific Inc.), according to the manufacturer’s protocol. The concentration of extracted protein was measured by the BCA method (Sigma-Aldrich Co. LLC). Equal amounts of protein samples were separated on a SDS-polyacrylamide gel (10–12%) and then transferred to a nitrocellulose membrane (Hybond ECL; Amersham Pharmacia Biotech Inc., USA) at 4 °C using Mini Trans-Blot^®^ cell system. Nitrocellulose membranes were blocked by 0.05% non-fat dried milk in deionized water for 1 hour. Subsequently, the nitrocellulose membranes were incubated overnight at 4 °C with specific primary antibodies at dilutions ranging from 1:500-1000. Antibodies specific for γ-H2AX (Ser139), PARP1, ATF3, p-ERK (Thr202 and Tyr204), JNK, p-p38 (Thr 180/Tyr 182), cleaved-lamin A/C and β-actin were purchased from Santa Cruz Biotechnology, Inc., p38, ERK, and cleaved-caspase3 antibodies were purchased from Cell Signaling Technology (Danvers, Massachusetts, USA), and anti-p-JNK (Thr 183/Tyr 185) was purchased from Millipore (Billerica, MA, USA). Unbound antibodies were removed from the nitrocellulose membranes in 5 PBST (0.05% Tween-20 in PBS) washes. The antibody-bound membranes were incubated for 2 hours with peroxidase-conjugated mouse or rabbit secondary antibodies diluted to 1:1000. Expression levels of proteins were detected using the SuperSignal West Pico ECL solution (Thermo Fisher Scientific Inc.) in Fuji LAS-3000 system (Fujifilm, Tokyo, Japan).

### Analysis of ROS generation in cytosol and in mitochondria

HK-2 cells (4 × 10^5^ cells/6 cm culture dish) were grown overnight and treated with ETO or ETO/NAC for 48 hours. MitoSox (5 μM; Invitrogen) and DCF-DA (10 μM; Invitrogen) were added for 10 and 15 minutes at 37 °C in an atmosphere of 5% CO_2_, respectively. The treated and untreated HK-2 cells were trypsinized for 3 minutes, washed in PBS and centrifuged at 200 × *g* for 3 minutes at 4 °C. After the supernatant was removed, PBS was added to the collected pellets and fluorescence intensity was analyzed using FACS Aria™ III with Diva™ software (BD Biosciences).

### Analysis of mitochondrial mass and respiration

HK-2 cells (4 × 10^5^ cells/6 cm culture dish) were grown overnight and treated with ETO or ETO/NAC for 48 hours. MitoTracker Red CMXRos and Green FM (100 nM; Invitrogen) were added for 10 minutes at 37 °C in an atmosphere of 5% CO_2_. Treated and untreated HK-2 cells were trypsinized for 3 minutes, washed with PBS and centrifuged at 200 × *g* for 3 minutes at 4 °C. The supernatant was removed, PBS was added to the collected pellets, and fluorescence intensity was analyzed using FACS Aria™ III with Diva™ software (BD Biosciences).

### Analysis of necrotic and apoptotic cell death

HK-2 cells (4 × 10^5^ cells/6 cm culture dish) were grown overnight and treated with ETO or ETO/NAC for 48 hours. Untreated and treated HK-2 cells were collected following a 3-minute incubation in trypsin-EDTA, then washed with PBS, and centrifuged at 200 × *g* for 3 minutes at 4 °C. Necrotic and apoptotic cell death were measured using FITC Annexin V Apoptosis Detection Kit I (BD Biosciences.), according to the manufacturer’s protocol. Fluorescence intensity was measured using FACS Aria™ III with Diva™ software (BD Biosciences.).

### Cytotoxicity analysis

The cells were grown overnight (seeded at 5 × 10^3^/well) in 96-well plates (BD Biosciences) and treated with indicated conditions. Cytotoxicity was measured using LDH Cytotoxicity Detection Kit (Takara Bio, Inc., Otsu, Shiga, Japan) according to the manufacturer’s protocol. The plates were read using microplate spectrophotometer (Molecular Devices) at 490 nm.

### Analysis of cell cycle

HK-2 cells (4 × 10^5^ cells/6 cm culture dish) were grown overnight and treated with ETO or ETO in combination with U0126 for 48 hours. Treated and untreated HK-2 cells were collected following a 3-minute incubation in trypsin-EDTA, then washed with PBS, and centrifuged at 200 × *g* for 3 minutes at 4 °C. The resuspended cells were added to a tube containing cold ethanol (70%) and PBS (30%), and were fixed overnight at 4 °C. The next day, the cells were washed with PBS (1 ml) and centrifuged at 200× *g* for 3 minutes at 4 °C. The resuspended cells were supplemented with PI (50 μg/ml; Sigma-Aldrich Co. LLC) containing RNase A (500 μg/ml; Sigma-Aldrich Co. LLC) and then analyzed using FACS Aria™ III with Diva™ software (BD Biosciences).

### Analysis of mtDNA copy number and mRNA level

HK-2 cells (4 × 10^5^ cells/6 cm culture dish) were grown overnight and treated with ETO or ETO/NAC for 48 hours. Total genomic DNA and RNA were extracted using QuickGene SP kit, including DNA tissue kit and RNA cultured cell kit (Fujifilm), according to the manufacturer’s protocol; and the concentrations were measured by Micro UV-Vis fluorescence spectrophotometer (Malcom, Tokyo, Japan). The mitochondrial DNA copy number and mRNA levels were measured by real-time PCR (Rotor-Gene Q; Qiagen, Valencia, CA, USA) using a QuantiTect SYBR Green PCR Kit. Primers were specific for ND1 and ND4 (mitochondrial DNA), and *TFAM, PGC-1α, PGC-1β*, and *GAPDH* (mRNA) as described previously^59^. Specific details for real-time PCR conditions and analysis have already been published^5^.

### Analysis of cytosolic ATP

HK-2 cells (4 × 10^5^ cells/6 cm culture dish) were grown overnight and treated with ETO or ETO/NAC for 48 hours. Untreated and treated cells were trypsinized for 3 minutes, washed by PBS, and centrifuged at 200 × *g* for 3 minutes at 4 °C. Cells were counted on a hematology analyzer (Paul Marienfeld GmbH & Co., KG, Bad Mergentheim, Germany) and reseeded (1 × 10^4^/well) into a 96-well plate (Greiner Bio-One.). The cytosolic ATP level was detected using a CellTiter-Glo^®^ Luminescent cell viability assay kit (Promega Corporation., Madison WI, USA) and quantified by Molecular Devices SpectraMax GEMINI fluorescence micro plate reader (Molecular Devices Inc.).

### Analysis of live cell image

HK-2 cells (4 × 10^5^ cells/6 cm culture dish) were grown overnight and treated with ETO or ETO/NAC for 48 hours. Cell morphology was observed using a phase contrast microscope (E-scope i304, Macrotech, Seoul, South Korea) and analyzed using the ScopePhoto software package.

### Analysis of caspase 3/7 activity

HK-2 cells (4 × 10^5^ cells/6 cm culture dish) were grown overnight and treated with ETO or ETO in combination with U0126 for 48 hours. Untreated and treated cells were trypsinized for 3 minutes, washed in PBS, and centrifuged at 200 × *g* for 3 minutes at 4 °C. The cells were resuspended and counted with a hematology analyzer (Paul Marienfeld GmbH & Co.) and then reseeded (1 × 10^4^/well) into a 96-well plate (Greiner Bio-One.). The caspase 3/7 activity was measured by using the Caspase-Glo^®^ 3/7 Assay (Promega Corporation) and Molecular Devices SpectraMax GEMINI fluorescence micro plate reader (Molecular Devices Inc.). The luminescence intensity was measured by Fuji LAS-3000 system (Fujifilm.).

### Analysis of cell viability using xCELLigence system for real-time

The xCELLigence system (RTCA DP Analyzer, Roche Applied Science, Penzberg, Germany) composition of RTCA Resistor E-Plate 16 devices (ACEA Biosciences Inc., San Diego, CA, USA) was kept in an incubator system (humidified atmosphere 5% CO_2_ at 37 °C) overnight. The E-Plate 16 devices were filled with RPMI1640 medium (100 μl) containing 1% penicillin/streptomycin and 10% FBS (Thermo Fisher Scientific Inc.) and measured using RTCA DP software (version 1.2) with background normalization. HK-2 cells (4 × 10^5^ cells/6 cm culture dish) were grown overnight and treated with ETO or ETO in combination with U0126 for 48 hours. Treated and untreated HK-2 cells were trypsinized for 3 minutes, washed with PBS and centrifuged at 200 × *g* for 3 minutes at 4 °C. Subsequently, these cells were counted by a hematology analyzer (Paul Marienfeld GmbH & Co.) and reseeded [1 × 10^5^/well in RPMI1640 medium (100 μl)] on E-Plate 16 devices connected to the xCELLigence system overnight. The next day, HK-2 cells were pre-treated with U0126 (20 μM) for 1 hour and then treated with etoposide (50 μM). Cell viability was measured using RTCA DP software (version 1.2) at 1-hour intervals for 48 hours.

### Analysis of nucleus swelling and NE topography using a hemocytometer and staining with Hoechst 33258 and PI

Extracted nuclei were seeded on a hemocytometer (Paul Marienfeld GmbH & Co.) and measured using phase contrast microscopy (E-scope i304, Macrotech Corporation). Analysis was performed using Scopephoto software. Extracted nuclei were also seeded on 24-well plates with a coverslip insert (SPL Life Sciences, Pochun, Korea) for 15 min at room temperature and then washed at least 3 times with PBS. After the nuclei were fixed with 3.7% formaldehyde for 15 min, they were stained with Hoechst 33258 (5 μM, 1:1000, Sigma-Aldrich Co. LLC) and PI (95 μM, 1:1000, Sigma-Aldrich Co. LLC) dyes for 15 min. The nuclei were measured using confocal microscopy (LSM-700, Carl Zeiss MicroImaging GmbH) and analyzed using the Zen 2009 software.

### Analysis of nuclear swelling and NE topography using AFM and staining with Hoechst 33258 and PI

HK-2 cells (4 × 10^5^ cells/6 cm culture dish) were grown overnight and treated with ETO or ETO in combination with U0126 for 48 hours. Treated and untreated cells were trypsinized for 3 minutes, washed with PBS, and centrifuged at 200 × *g* for 3 minutes at 4 °C. Cytoplasmic Extraction Reagent I (100 μl) containing protease and phosphatase inhibitor cocktails (1:100, Thermo Fisher Scientific Inc.) prepared in NE-PER^®^ Nuclear and Cytoplasmic Extraction Reagents (Thermo Fisher Scientific Inc.) were added to the samples for 10 minutes on ice (4 °C) during which the cells were gently tapped. Subsequently, Cytoplasmic Extraction Reagent II (5.5 μl) was added to the samples for 1 minutes on ice (4 °C) and samples were gently tapped. For extraction of nuclei, samples were centrifuged at 16,000 × *g* for 5 minutes at 4 °C and then resuspended in PBS (1 ml). The nucleus size was measured by hematology analyzer (iNCYTO, C-Chip DHC-N01, Chungcheongnam-do, South Korea) and observed by microscopy (Olympus IMT-2; Olympus Corporation, Tokyo, Japan). Extracted nuclei were seeded on 6 cm culture dish for 15 minutes. After, nuclei were fixed with formaldehyde (3.7%) for 15 minutes, washed with PBS and deionized water for three times, and measurements were obtained using microscopy (Olympus IMT-2; Olympus Corporation). For AFM height measurements, experiments were performed in non-contact mode using a Cypher (Asylum Research, Inc., Santa Barbara, USA) with Si cantilevers (270–310 kHz resonant frequency), and for NE topography analysis, the scanning rate was ∼0.4 Hz. AFM image analysis, consisting of 3D topography and measurements of line profile, were performed using Gwyddion software (http://gwyddion.net/). For confocal microscopy, extracted nuclei were also seeded on 24-well plates with a coverslip (SPL Life Sciences, Pochun, Korea) for 15 min at room temperature and then washed at least 3 times with PBS. After the nuclei were fixed with 3.7% formaldehyde for 15 min, they were stained with Hoechst 33258 (5 μM, 1:1000, Sigma-Aldrich Co. LLC) and PI (5 μM, 1:1000, Sigma-Aldrich Co. LLC) dyes for 15 min. The nuclei were measured using confocal microscopy (LSM-700, Carl Zeiss MicroImaging GmbH) and analyzed using the Zen 2009 software.

### Statistical analyses

The statistical analyses were performed by one-way ANOVA using the SigmaPlot software version 12.0 (Systat Software Inc., USA). All experiments were repeated independently at least three times. Statistical significance was defined as a P-value of *P < 0.05, **P < 0.01.

## Additional Information

**How to cite this article**: Shin, H.-J. *et al*. Etoposide induced cytotoxicity mediated by ROS and ERK in human kidney proximal tubule cells. *Sci. Rep.* **6**, 34064; doi: 10.1038/srep34064 (2016).
